# Supplementary material for: Versatile, marker-free platform for life cycle-wide imaging of Plasmodium falciparum by integrating an exogenous gene cassette into a conserved intergenic locus
Source: Sci Rep. 2026 Jun 18;16:27873. doi: 10.1038/s41598-026-55934-7 (PMC13547369; doi:10.1038/s41598-026-55934-7)
Supplement: Supplementary file 1 [file 41598_2026_55934_MOESM3_ESM.docx]

**Versatile, marker-free platform for life cycle-wide imaging of *Plasmodium falciparum* by integrating an exogenous gene cassette into a conserved intergenic locus**

Takashi Sekine^1^, Naoaki Shinzawa^1^#, Rie Kubota^1^, Daisuke Kobayashi^2^, Yawara Okubo^1^, Kentaro Itokawa^2,3^, Haruhiko Isawa^2^, Hisako Amino^1^, Tomoko Ishino^1^

^1^ Department of Parasitology and Tropical Medicine, Graduate School of Medical and Dental Sciences, Institute of Science Tokyo, Tokyo, Japan.

^2^ Department of Medical Entomology, National Institute of Infectious Diseases, Japan Institute for Health Security, Tokyo, Japan.

^3^ Antimicrobial Resistance Research Center, National Institute of Infectious Diseases, Japan Institute for Health Security, Tokyo, Japan.

#Address correspondence to Naoaki Shinzawa, [shinzawa.vip@tmd.ac.jp](mailto:shinzawa.vip@tmd.ac.jp)

**Contents:**

- **Supplementary Figure 1-4**
- **Supplementary Table 1 (Separate Excel file):** Whole-genome sequencing-based SNPs and indels in NF54-mCh
- **Supplementary Table 2 (Separate Excel file):** Oligonucleotides used in this study

**Supplementary Figure 1. Confirmation of the loss of the plasmid.** PCR was performed using a primer set designed to target the *yfcu* coding region on the plasmid. The original gel image is presented in Supplementary Figure 4.

**Supplemental Figure 2. Detection of oocysts using mercurochrome.** Mercurochrome-staining of the NF54 midgut. The midguts were dissected at 8-10 dpi. A scale bar indicates 100 µm.

**Supplemental Figure 3. mGreenLantern fluorescence throughout the life cycle of *P. yoelii*.** (A) mGreenLantern expression during asexual development and gametocytes in Py-mGL. Thin smears of Py-mGL infected erythrocytes were made without fixation, and mGreenLantern fluorescence (green, top panels) was captured by a fluorescent microscope. All mGreenLantern images were captured with the same exposure time. Nuclei were stained with Hoechst (cyan, middle panels). Phase images are shown below. The bar indicates 10 µm.

(B) A representative image of mGreenLantern expression oocysts on the midgut of Py-mGL infected *A. stephensi* mosquito. A midgut was dissected at day 10 post-feeding and observed by a fluorescent microscope with a phase image. The bar indicates 100 µm.

(C) Micrographs of a sporozoite-containing salivary gland. The salivary gland of Py-mGL infected mosquito was collected at day 20 post-feeding and stained with Hoechst to visualize gland nuclei (blue) and CellMask^TM^ Orange to visualize plasma membranes (red). Fluorescent images are shown as max-intensity projections of confocal stacks. The bar indicates 100 µm.

**Supplementary Figure 4.** **Uncropped images of gels and blots.** Dashed boxes indicate the regions presented in the corresponding figures.
